# IFT20 and WWTR1 govern bone homeostasis via synchronously regulating the expression and stability of TβRII in osteoblast lineage cells

**DOI:** 10.21203/rs.3.rs-4009802/v1

**Published:** 2024-03-19

**Authors:** Yang Li, Shuting Yang, Shuying Yang

**Affiliations:** 1 Department of Basic & Translational Sciences, School of Dental Medicine, University of Pennsylvania, Philadelphia, PA 19104, USA; 2 Department of Orthopaedic Surgery, School of Medicine, Johns Hopkins University Baltimore, MD 21205, USA; 3 Department of Periodontics, School of Dental Medicine, University of Pennsylvania, Philadelphia, PA 19104, USA; 4 The Penn Center for Musculoskeletal Disorders, School of Medicine, University of Pennsylvania, Philadelphia, PA 19104, USA

**Keywords:** IFT20, WWTR1, bone homeostasis, TβRII, osteoblasts

## Abstract

Balance of bone and marrow fat formation is critical for bone homeostasis. The imbalance of bone homeostasis will cause various bone diseases, such as osteoporosis. However, the precise mechanisms governing osteoporotic bone loss and marrow adipose tissue (MAT) accumulation remain poorly understood. By analysis of publicly available databases from bone samples of osteoporosis patients, we found that the expression of intraflagellar transport 20 (IFT20) and WW domain containing transcription regulator 1 (WWTR1) were significantly downregulated in osteoblast lineage cells. Additionally, we found that double deletions of IFT20 and WWTR1 in osteoblasts resulted in a significant accumulation of MAT and bone loss. Moreover, IFT20 and WWTR1 deficiency in osteoblasts exacerbated bone-fat imbalance in ovariectomy (OVX)- and high-fat-diet (HFD)-induced osteoporosis mouse models. Mechanistically, we found that deletions of IFT20 and WWTR1 in osteoblasts synergistically inhibited osteogenesis and promoted adipogenesis and osteoclastogenesis. We also found that IFT20 interacted with TGF-β receptor type II (TβRII) to enhance TβRII stability by blocking c-Cbl-mediated ubiquitination and degradation of TβRII. WWTR1 transcriptionally upregulated TβRII expression by directly binding its promoter. These findings indicate that targeting IFT20/WWTR1 may be a potential therapeutic strategy for the treatment of osteoporosis.

## Introduction

Osteoporosis (OP) is a metabolic bone disease caused by environmental and genetic factors at any age and gender.^1,2^ Osteoporosis has risen rapidly in the US, with the direct cost reaching ~17.5 billion dollars annually.^3^ Due to the enormous social, economic, and healthcare impact, osteoporosis is a pressing health issue requiring effective therapeutic strategies. It is well known that bone marrow is the only tissue where bone cells and fat coexist.^4^ Bone homeostasis is strictly orchestrated by multiple molecular signals and cues from the microenvironment of bone marrow.^5–7^ The imbalance of bone homeostasis could cause osteoporosis.^8,9^ In the bone marrow cavity of osteoporosis patients, the proliferation and differentiation of osteoblasts are inhibited, but MAT accumulation is increased.^10–12^ In mice, HFD not only leads to obesity but also causes bone loss and osteoporosis with raised MAT in the bone marrow.^13,14^ Numerous studies also showed that MAT accumulation usually increases at the expense of bone formation,^4,13^ demonstrating a close connection between bone and fat in the bone marrow. However, the precise mechanisms governing osteoporotic bone loss and MAT accumulation remain poorly understood.

Osteoblasts play critical roles in maintaining the functional skeletal systems by regulating osteogenetic markers’ expression, such as runt-related transcription factor 2 (Runx2), osterix (OSX), and osteocalcin (OCN).^15,16^ Dysfunction of osteoblasts could result in various bone diseases, including osteoporosis.^17,18^ Moreover, disruption of the balance between adipogenesis and osteogenesis has been reported to contribute to osteopenia accompanied by progressive marrow adiposity. Nevertheless, what signaling molecules or critical genes control the commitment and function of osteoprogenitor cells are still undiscovered.

Ciliary protein IFT20 is essential for craniofacial skeletal development and bone formation.^19,20^ Our previous studies showed that IFT20 governs the fate of mesenchymal stem cells (MSCs) by promoting osteogenesis and inhibiting adipogenesis;^21^ deletion of IFT20 in osteoblasts impairs the bone microarchitecture and reduces the bone mass but has no apparent effect on MAT in mice,^21,22^ suggesting that IFT20 alone mainly functions in osteogenesis. WWTR1 is a critical fate-determinant factor in MSCs.^23,24^ It can interact with multiple transcription factors, including Runx2 and peroxisome proliferator-activated receptor γ (PPARγ), to enhance osteoblast differentiation and repress adipocyte differentiation by promoting Runx2 and inhibiting PPARγ transcription.^24–26^ Notably, global knockout of WWTR1 in mice displayed bone loss and shorter cilia in cystic epithelia by downregulating ciliary proteins such as kinesin family number 3A (Kif3a) and polycystin-1 (Pkd1).^27,28^ Moreover, compound-heterozygous loss of Pkd1 and WWTR1 are additive in osteogenesis and adipogenesis compared to the single deficiency of Pkd1 or IFT20.^25^ Most notably, IFT20 and WWTR1 were identified as fatal drivers for the pathogenesis of cystic kidney disease in clinics,^27,29^ implying that the two proteins may have a correlation or association in functions or molecular mechanism regulation. By screening the significant genes from patients with osteoporosis, we found that the expression of IFT20 and WWTR1 significantly decreased. These findings strongly suggested that IFT20 and WWTR1 may synchronously regulate bone homeostasis.

In this study, we characterized the contribution of IFT20 and WWTR1 to bone homeostasis and determined the molecular mechanisms of IFT20/WWTR1-droved bone homeostasis by conditional knock-out of IFT20 and WWTR1 in osteoblasts. Our data revealed a new mechanism that IFT20 and WWTR1 can synchronously govern the expression and stability of TβRII to inhibit MAT accumulation during bone development. Decrease or loss of IFT20 and WWTR1 accelerated MAT accumulation even at the young age of mice. These findings demonstrated that IFT20 and WWTR1 are critical for controlling marrow fat and bone homeostasis.

## Results

### IFT20 and WWTR1 signatures are decreased in osteoblast lineage cells derived from human and mouse osteoporotic bone.

To investigate the potential role of IFT20 and WWTR1 on bone homeostasis and diseases, we first identified the expressions of IFT20 and WWTR1 by analyzing publicly available expression data of osteoporosis patients (GSE35958).^30^ Our data revealed that IFT20 and WWTR1 expressions are significantly decreased in osteoblast lineage cells from the bone of osteoporotic patients compared to the controls (Fig. 1a). To better understand the role of IFT20 and WWTR1 in pathological bone loss, we examined their expressions in the OVX- and HFD- induced osteoporotic mouse models. In line with the human data, the signatures of IFT20 and WWTR1 were significantly decreased in primary osteoblasts from calvaria of post-6-week-OVX mice (Fig. 1b) and HFD-induced osteoporotic bone loss mouse model (Fig. 1c) compared to those in the controls, suggesting that IFT20 and WWTR1 may be critical regulators in osteoporosis.

### Double deletions of IFT20 and WWTR1 in osteoblasts cause a severe osteoporosis-like phenotype.

To investigate the function of IFT20 and WWTR1 in bone formation and homeostasis, we generated three gene conditional knockout lines with single and double deletions of IFT20 and WWTR1 in osteoblasts by breeding IFT20 and WWTR1 single and double floxed lines with osterix (OSX) Cre transgenic mice (hereafter named IFT20-KO, WWTR1-KO and dKO). RT-PCR verified that their expressions were efficiently extinguished in primary osteoblasts (Supplementary Fig. 1a, b). Intriguingly, we found that double deletions of IFT20 and WWTR1 in osteoblasts resulted in additive bone loss and displayed a severe osteoporosis-like phenotype compared to the single deletion of IFT20 or WWTR1 in osteoblasts (Fig. 2a), as evidenced by the 41%, 12.04% and 20.3% reduction in the bone mineral density (BMD) in IFT20-KO and WWTR1-KO, dKO mice compared to the controls (OSX-Cre mice) (Fig. 2b). Consistently, we found the femurs from dKO mice lost approximately 50% of bone volume per total volume (BV/TV) (Fig. 2c). Histomorphometry analysis of femur metaphysis showed that loss of IFT20 and WWTR1 in osteoblasts resulted in significant bone loss in the trabecular bones compared to the age-matched controls (Fig. 2d–f). To further identify the function of IFT20 and WWTR1 in osteogenesis, we isolated primary osteoblasts from calvaria of IFT20 and WWTR1 double and single deficient mice and controls to characterize their osteogenic potentials *in vitro*. As expected, we found the alkaline phosphatase (ALP) activity was significantly decreased in dKO mice (Fig. 2g) after stimulation with osteogenic induction media for 5 days. Consistently, the mineralized nodule formation (ARS) also displayed a significant decrease in dKO mice after stimulation with osteogenic induction media for 2 weeks (Fig. 2h, i). Moreover, we found that deletions of IFT20 and WWTR1 in primary osteoblasts significantly decreased the expression of osteogenic markers such as Runx2, OSX, and OCN (Fig. 2j). Overall, these data suggested that IFT20 and WWTR1 in osteoblasts can synergistically regulate bone formation.

### IFT20 and WWTR1 deficiency in osteoblasts decreases bone formation and increases MAT accumulation.

Previous studies showed osteoporosis is a primary metabolic bone disease in which bone loss is accompanied by MAT accumulation and impaired osteoclastogenesis.^4,13^ As such, we explored whether IFT20 and WWTR1 deficiency in osteoblasts affect MAT accumulation. Histological examination revealed a noticeable trabecular bone loss accompanied by a significant increase in the number and density of marrow adipocytes in the bone marrow milieu of dKO mice compared to the controls. In contrast, only a few adipocytes were observed in IFT20-KO and WWTR1-KO mice (Fig. 3a–c). These results were further confirmed by micro-computed tomography (μCT) results, as evidenced by a significantly increased fat droplet in decalcified tibiae stained with osmium tetroxide (OsO4) (Fig. 3d, e). In addition, the results from tartrateresistant acid phosphatase (TRAP) staining showed that TRAP^+^ osteoclasts were significantly enhanced in the dKO mice compared to the single deletion of IFT20 or WWTR1 in osteoblasts and the control group (Fig. 3f, g), suggesting that decreased bone mass may be partly due to increased osteoclastogenesis and adipogenesis.

### IFT20 and WWTR1 synchronously activate TβRII signaling to promote osteogenesis.

By analysis of the human osteoporosis database GSE35958, the Kyoto Encyclopedia of Genes and Genomes (KEGG) data revealed that the transforming growth factor β (TGF-β) signaling pathway exhibited a conserved signature as one of the significantly top enriched gene sets (Fig. 4a). Next, we identified the expression of TβRI and TβRII in primary osteoblasts from dKO mice and age-matched controls. Interestingly, we found that TβRII expression was significantly decreased, but TβRI had no significant change (Fig. 4b). To further investigate the potential effect of IFT20 and WWTR1 deficiency in osteoblasts on TβRII downstream signaling, we analyzed the critical proteins downstream TGF-β signaling pathway such as TβRII, pSmad2/3, and Smad2/3. Western blot analysis showed that double depletions of IFT20 and WWTR1 in primary osteoblasts significantly reduced the expression of TβRII and pSmad2/3 compared to the single deletion (Fig. 4c). Consistently, immunofluorescence staining showed a significant decrease in Smad2/3 nuclear translocation in IFT20/WWTR1-mutant osteoblasts (Fig. 4d). These results suggested that IFT20 and WWTR1 synchronously promote the TGF-β signaling pathway. To further examine whether the defective osteogenesis directly results from decreased expression of TβRII caused by IFT20/WWTR1 mutation, we overexpressed TβRII in IFT20/WWTR1-mutant osteoblasts and identified the osteogenic differentiation (Fig. 4e and supplementary Fig. 2). As expected, we found that the restoration of TβRII expression restored osteogenic differentiation in IFT20/WWTR1-mutant osteoblasts (Fig. 4e).

### IFT20 inhibits the ubiquitination and degradation of TβRII via c-Cbl.

Previous findings demonstrated that loss of IFT20 in fibroblasts promoted the autoubiquitination and proteasomal degradation of E3 ubiquitin ligase c-Cbl.31 Moreover, TβRII stability was inhibited by knockout of c-Cbl in mouse embryonic fibroblasts. In contrast, overexpression of c-Cbl improved the stability of TβRII and sensitization of cells to TGF-β.32 Given these findings, we speculated that IFT20 might regulate TβRII stability through c-Cbl signaling. After treatment of protein synthesis inhibitor cycloheximide for 48 hrs, we found that overexpression of IFT20 in primary osteoblasts significantly increased TβRII stability compared to the control groups (Fig. 5a). Fabian Marc Schmid *et al*. reported that IFT20 could interact with c-Cbl to inhibit its autoubiquitination and degradation.^31^ To further characterize the function of IFT20 in TβRII stability, we overexpressed IFT20 and/or TβRII in the primary osteoblasts. Co-IP data showed that IFT20 could bind to TβRII protein directly in the primary osteoblasts (Fig. 5b), indicating that IFT20 may be involved in c-Cbl-mediated the stability of TβRII protein in osteoblasts. To test whether IFT20 affects TβRII stabilization through the c-Cbl, we co-transfected HA-Ub with/without flag-TβRII and GFP-IFT20 in osteoblasts. Western blot results revealed that TβRII ubiquitination was markedly inhibited after overexpression of IFT20 (Fig. 5c). Moreover, we found a significant decrease in c-Cbl expression due to the loss of IFT20 in osteoblasts (Fig. 5d). Our data also showed that overexpression of IFT20 in primary osteoblasts improved TβRII stability by inhibiting its ubiquitination; however, after silence of c-Cbl by siRNA, the stability of TβRII was significantly interfered and could be partly reversed in IFT20-overexpressed group (Fig. 5e). In addition, we found that overexpression of IFT20 in c-Cbl-mutant osteoblasts led to a decreased ubiquitination of TβRII compared to the controls (Fig. 5e). Collectively, these data demonstrated that IFT20 inhibits the ubiquitination and degradation of TβRII via the c-Cbl-mediated proteasome pathway.

### WWTR1 is required for the endogenous expression of TβRII.

Emerging evidence revealed that WWTR1 is a positive regulator of TGF-β signaling by promoting nuclear translocation of Smad2/3.^33,34^ Because double deletions of IFT20 and WWTR1 pronouncedly decreased TβRII levels compared to the single deletion of IFT20 or WWTR1, suggesting that increased TβRII level was not only caused by TβRII stability regulated by IFT20. TEA domain transcription factor 1 (TEAD1) functions as an essential transcriptional co-activator of WWTR1 to regulate downstream regulators’ expression due to lack of direct DNA binding domain of WWTR1.^35–37^ After silencing TEAD1 by *TEAD1* siRNA in primary osteoblasts from WWTR1-KO mice, we found that the expression of TβRII was significantly decreased compared to the control group OSX-Cre (Fig. 6a), suggesting that the complex of WWTR1/TEAD1 plays a critical role in the regulation of TβRII. To further confirm the function of the WWTR1/TEAD1 complex in the regulation of TβRII, we analyzed the binding site of the WWTR1/TEAD1 complex in the *TβRII* promoter using the Vector NTI software. As expected, we found a crucial WWTR1/TEAD1 complex binding site in the *TβRII* promoter region (Fig. 6b). Therefore, we examined whether WWTR1 regulates TβRII expression directly in primary osteoblasts. As expected, our data showed that the TβRII transcriptional activity was significantly inhibited due to loss of WWTR1 in osteoblasts (Fig. 6c). After silencing TEAD1 by *TEAD1* siRNA in osteoblasts from WWTR1-KO mice, the *TβRII* transcriptional activity was significantly suppressed (Fig. 6d). Taken together, our data demonstrated that IFT20 and WWTR1 synchronously regulated osteoblast differentiation via modulating TβRII expression and protein stability. IFT20 enhanced the stability of TβRII by blocking c-Cbl-mediated ubiquitination and degradation of TβRII; WWTR1 increased TβRII expression by binding its promoter directly (Fig. 6e).

### IFT20 and WWTR1 deficiency in osteoblasts exacerbate bone-fat imbalance in OVX- and HFD-induced osteoporosis.

To better understand the role of IFT20 and WWTR1 in pathological bone loss, we examined the influence of IFT20 and WWTR1 deficiency in osteoblasts on osteoporotic bone loss in mice following OVX and HFD feeding. At week 6 following OVX, we found that dKO mice showed approximately 75% lower BV/TV compared to that in the sham group, suggesting that IFT20 and WWTR1 deficiency in osteoblasts exacerbated OVX-induced osteoporosis (Fig. 7a, b). The results from H&E staining of the femurs showed that the number and area of adipocytes were significantly increased in dKO mice compared to those in OSX-Cre mice after OVX (Fig. 7c, d). To further confirm the effect of IFT20 and WWTR1 deficiency in osteoblasts on MAT formation, we conducted the μCT analysis of OsO4 staining of decalcified tibiae from dKO mice and controls following OVX. The dKO mice displayed a dramatic increase in the regulated MAT (rMAT) compared to the controls (Fig. 7e, f). Recent studies showed that HFD is considered as a crucial environmental factor in reducing bone mass and promoting MAT expansion.^13^ To further corroborate the role of IFT20 and WWTR1 in the regulation of bone-fat balance and bone metabolism, we identified the contribution of IFT20 and WWTR1 deficiency in osteoblasts to HFD-induced MAT expansion and bone loss. As expected, we found that HFD significantly reduced the bone mass of trabecular bones in dKO mice compared to age-matched controls (Fig. 7g, h). Consistently, the rMAT expansion in the bone marrow of the tibia from the dKO mice was significantly enhanced (Fig. 7I, J), further supporting that IFT20 and WWTR1 synchronously regulate bone and marrow fat homeostasis.

## Discussion

At birth, bone marrow is mainly composed of hematopoietic cells, known as red marrow. MAT increases dramatically during postnatal growth, causing the change of marrow color from red to yellow.^38^ However, how marrow fat and bone homeostasis are finely regulated remains largely unknown. Osteoblasts are essential for bone homeostasis and regeneration.^39–41^ Dysfunction of osteoblasts could delay bone formation and cause a spectrum of diseases, such as osteoporosis and dwarfism.^17,42^ Here, by combined deletions of IFT20 and WWTR1 in osteoblasts using OSX-Cre mice, we found for the first time that loss of IFT20 and WWTR1 in osteoblasts significantly increased MAT accumulation and reduced bone mass, showing a severe osteoporosis-like phenotype in 12-week-old dKO mice. Mechanistically, we revealed that IFT20 and WWTR1 play essential roles in osteoblasts for controlling MAT and bone homeostasis via regulating the stability and expression of TβRII. Thus, this study reveals new regulators and molecular mechanisms of bone-fat balance and provides a genetic and molecular basis for developing new strategies and drugs for osteoporosis and other bone-fat-related diseases.

Evidence reveals that ciliary proteins IFT play critical roles in regulating adipogenesis and osteogenesis.^21,25^. Deletion of ciliary proteins such as Kif3a and IFT80 in osteoblasts led to osteoporosis; in contrast, deletion of Kif3a and other ciliary proteins in adipocytes enhanced lipid accumulation by improving PPARγ expression.^25,43–46^ Previous studies also showed that forced overexpression of WWTR1 in osteoblast lineages significantly increased bone mass and reduced adipogenesis.^35,47^ Moreover, global knockout of WWTR1 in mice exhibits ossification defects,^27^ and depletion of WWTR1 in zebrafish impairs bone development.^35^ Supportively, our data showed that double deletions of IFT20 and WWTR1 could synergistically promote MAT formation and bone loss compared to single deletion of IFT20 or WWTR1 in osteoblasts, suggesting that both proteins are essential for the maintenance of marrow adipocyte and osteoblast formation. Decreased expression of IFT20 and/or WWTR1 contributes to MAT formation and bone homeostasis during postnatal development stages. Interestingly, we also found that OVX and HFD can significantly enhance MAT formation and bone loss in dKO mice, demonstrating the importance of the synergistic role of these two proteins during aging caused bone loss process.

Bone homeostasis is tightly regulated by adipocytes besides osteoclasts and osteoblasts.^9,48^ Our previous findings and others demonstrated that the bone marrow adipocytes could secret receptor activators of nuclear factor kappa-B ligand (RANKL) to induce osteoclastogenesis and inhibit bone formation.^11,21^ Interestingly, we found the osteoclast numbers were significantly increased after deletions of IFT20 and WWTR1 in osteoblasts using OSX-Cre compared to the single knockout of IFT20 or WWTR1 in mice, suggesting that bone loss was partly caused by increased osteoclastogenesis in dKO mice, reinforcing our findings that IFT20 and WWTR1 synergistically govern the marrow fat and bone homeostasis via affecting osteoblasts, adipocytes, and osteoclasts. Furthermore, some studies have demonstrated that adipocytes control bone mass and that the ablation of adipocytes enhances bone formation.^49,50^ These results further support the tenet that IFT20 and WWTR1 regulate bone-fat balance in a context-specific manner.

TGF-β signaling has emerged as a critical signaling, coordinating cell proliferation and differentiation to regulate bone homeostasis by activating TβRII.^51–53^ Deletion of TβRII in osteoblasts dramatically decreased bone mass and increased MAT amount.^54^ In our previous study, by analysis of RNA-seq data from IFT20-deficient osteoblasts, we found that loss of IFT20 in these cells inhibited TGF-β signaling.^21^ Recent findings showed that TβRII stability was regulated by c-Cbl.^32^ Global knockout of c-Cbl in mice delayed bone development,^55,56^ and knockout of c-Cbl in fibroblasts decreased TβRII stability and desensitized the cells to TGF-β stimulation.^32^ In contrast, c-Cbl overexpression stabilizes TβRII and sensitizes leukemia cells to TGF-β. Moreover, the decreased TβRII caused by c-Cbl knockdown could be restored by the ectopic expression of c-Cbl.^32^ This evidence strongly supported our findings that IFT20 is a crucial protein in regulating TβRII stability via c-Cbl regulation. Besides these, IFT20 was also reported to negatively regulate the autoubiquitination and proteasomal degradation of c-Cbl in fibroblasts,^31^ demonstrating the close regulation between IFT20, c-Cbl, and TβRII. WWTR1 can regulate TGF-β signaling by regulating the nucleocytoplasmic shuttling of Smad2/3.^33,34,57^ Interestingly, we found that loss of WWTR1 in osteoblasts inhibited TGF-β signaling by downregulating pSmad2/3 expression. Additionally, our data also showed that WWTR1 can significantly upregulate TβRII expression by directly binding its promoter. Thus, IFT20 and WWTR1 could govern bone formation and inhibit marrow fat accumulation via synchronously regulating the expression and stability of TβRII in osteoblasts.

## Materials and Methods

### Animals

OSX-Cre and floxed IFT20 mice were purchased from the Jackson Laboratory (Bar Harbor, MA, USA). The floxed WWTR1 mice were generated by our lab. For the OVX-induced osteoporosis mouse model, 3-month-old female dKO mice and age-matched controls (OSX-Cre) were subjected to sham and OVX surgery after anesthesia. At week 6 following OVX surgery, the bone mass and MAT were analyzed as previously reported.^13,21^ For the HFD-induced osteoporosis mouse model, 1-month-old male dKO mice and control littermates were fed high-diet fat for 3 months and then were euthanized for analysis. The animals were maintained according to the Animal Care and Use Committee of the University of Pennsylvania.

### Reagents, and plasmids

Antibodies against Smad2/3 and pSmad2/3 antibodies were obtained from Cell Signaling Technology (CST, USA). Antibodies against TEAD1, TβRII, GFP, flag, Ub, GAPDH, *c-Cbl* siRNA, and *TEAD1* siRNA were ordered from Santa Cruz Biotechnology. The secondary fluorescent antibodies and H&E staining kit were ordered from Abcam. Calcein labeling, osmium tetroxide, and lipofectamine^™^ 3000 were purchased from Fisher Scientific^™^. Dexamethasone, L-ascorbic acid, and β-glycerophosphate were ordered from Sigma. Plasmids pcDNA3.1-flag and pcDNA3.1-flag-TβRII, GFP-IFT20, HA-Ub were obtained from Addgene.

### Cell culture

Primary osteoblasts were isolated from the calvaria of IFT20-KO, WWTR1-KO, dKO mice, and controls, as described before.^45^ Briefly, the fresh calvaria from these mice above were cut into pieces and subjected to an enzyme solution containing 1 mg/ml collagenase type I and collagenase type II, collected, and cultured in α-Minimum Essential Medium (α-MEM; Gibco, USA) containing1×Pen-Strep solution (Fisher Scientific^™^, USA) and 10% fetal bovine serum (FBS; Gibco, USA) and at 37°C with 5% humidified CO2.

### ALP activity and osteogenic differentiation

The primary osteoblasts from calvaria of dKO mice and age-matched controls were cultured in osteogenic medium (α-MEM supplemented with 100 nM dexamethasone, 50 μg/mL L-ascorbic acid, 5 mM β-glycerophosphate). After induction for 5 days by osteogenic medium, the cells were harvested and incubated with the assay buffer (100 mM glycine (pH 10.5), and 1 mM MgCl_2_, 50 mM p-nitrophenyl phosphate solution) for 15 min at 37°C. Then, the reaction was stopped by stop solution (0.1 N NaOH) and measured at OD_405_ nm by a microplate reader, as we previously reported.^21^

For osteogenic differentiation, the primary osteoblasts from calvaria of IFT20-KO, WWTR1-KO, dKO mice, and age-matched controls were induced with osteogenic media for 2 weeks. After fixing for 2 mins with 4% PFA solution at room temperature, the cells were stained by the Alizarin Red S staining solution and analyzed as we previously reported.^21,45^

### qRT-PCR and ChIP-qPCR

The total RNA was extracted from primary osteoblasts using TRIzol reagent (TaKaRa, Japan). The cDNA was reverse transcribed using PrimeScript^™^ RT Kit (TaKaRa, Japan). Then, the qRT-PCR was carried out with the SYBR Green mixture. For ChIP-qPCR, the experiments were carried out using the Imprint Chromatin Immunoprecipitation Kit (Sigma, USA), as we previously reported.^35,58^ The primers in this study were listed in the Supplementary Table S1.

### Western blot

The primary osteoblasts as indicated lysed with modified RIPA buffer (Thermo Fisher Scientific, USA) were harvested and subjected to SDS-PAGE gels (Bio-Rad, USA). After PVDF membrane transference (Millipore, USA), the membranes were immunoblotted with the primary antibodies overnight at 4°C, washed by 3 times with TBST (0.1% Tween-20 in Tris-buffered Saline), incubated with HRP-conjugated secondary antibody for 1 hr at room temperature, and analyzed by ECL solution (Thermo Fisher, USA) as we previously described.^35,58,59^

### Immunofluorescence

The primary osteoblasts from the calvaria of dKO mice and controls were seeded in the glass coverslips. After culturing for 48 hr, the primary osteoblasts were fixed by 4% PFA and washed by 0.1% Triton X-100 in Tris-buffered Saline (TBST) 3 times. The cells were blocked by 1% bovine serum albumin (BSA) for 1 hr at room temperature and incubated with pSmad2/3 antibody (1:200 dilution) overnight at 4°C.

After washing 3 times with TBST, the cells were incubated with fluorescent antibody (1:1000 dilution) and DAPI for 1 hr at room temperature. Then, the cells were washed 3 times with TBST and visualized under a fluorescence microscope, as we previously reported.^21,35,58^

### Calcein labeling

Calcein (20 mg/kg) was subjected to 3-month-old IFT20-KO, WWTR1-KO, dKO mice, and age-matched controls on Day 2 and Day 5 before sacrifice through intraperitoneal injections. After sacrifice, the tibiae from these mice above were collected and fixed with 4% PFA solution at 4°C. After washing 3 times, the tibiae were infiltrated in 10% potassium hydroxide solution for 3 days at 4°C. And then, the tibiae were gradually dehydrated by 70%, 80%, 95%, 100% ethanol and xylene. Finally, the tibiae were embedded in the paraffin, sectioned, and observed by the fluorescence microscope. The Leica microanalysis system analyzed MAR and BFR, as we previously reported.^21,35^

### Histology

Briefly, the femurs from IFT20-KO, WWTR1-KO, dKO mice, and age-matched controls were harvested and fixed in 4% PFA solution overnight at 4°C. After decalcification with 10% ethylenediaminetetraacetic acid (EDTA) in PBS (pH 7.4) for 1 month, the femurs were embedded in paraffin and then sectioned. H&E and TRAP staining was carried out using the H&E staining kit (Abcam, USA) and TRAP staining kit (Sigma, USA), respectively, as we previously reported.^21,35,60^

### Radiographic analysis

μCT analysis of the femurs from 3-month-old IFT20-KO, WWTR1-KO, dKO, OVX- and HFD-induced mice and age-matched controls were performed and analyzed by a high-resolution μCT system as we described previously.^35,58–60^ For OsO4 MAT analysis, the whole intact tibiae of dKO mice, OVX- and HFD-induced mice and age-matched controls were dissected, fixed in 4% PFA solution for 48 hrs, and decalcified in 10% EDTA for 1 month. Then, the tibiae from these mice were treated with 2% OsO4 solution for 2 hrs at room temperature. After washing for 2 days by ddH2O, the bones were analyzed by a high-resolution μCT system. Quantification of fat volume, density, and distribution throughout the marrow was registered to the low contrast decalcified bone, as we previously described.^21,35^

### Statistical analysis

The publicly available data from the patients with osteoporosis from GSE35958^30^ were analyzed by R. Experimental data in this study was conducted and analyzed by GraphPad Prism 9. The data were reported as mean ± SEM by the Student’s t-test. The statistical significance of group differences was determined wby the 2-way ANOVA.

## Figures and Tables

**Fig. 1.** IFT20 and WWTR1 signatures are decreased in human and mouse samples with osteoporosis. **a** A Volcano plot of transcriptome profiles in osteoblast linage cells from the patients with osteoporosis (GSE35958). **b, c** Relative mRNA level of *IFT20* and *WWTR1* in primary osteoblasts from the calvaria of the OVX- (**b**) or HFD-induced (**c**) osteoporotic mice. N=3. **P < 0.01, ***P < 0.001.

**Fig. 2.** Double deletions of IFT20 and WWTR1 in osteoblasts cause a severe osteoporosis-like phenotype. **a** Representative μCT images of femurs from 3-month-649 old IFT20-KO, WWTR1-KO, dKO mice and age-matched controls. Scale bars, 100 μm. N=5. **b, c** Quantitative measurements of BMD and BV/TV of distal femurs (**a**). **d** Representative images of calcein double labeling proximal tibias from 3-month-old IFT20-KO, WWTR1-KO, dKO mice, and age-matched controls. Scale bar, 20 μm. N=5. **e, f** Quantification of bone formation rate (BFR) and mineral apposition rate (MAR) from (**d**) as indicated. N=5. **g** ALP activity in primary osteoblasts after stimulation with osteogenic media for 5 days as indicated. **h** Representative images of ARS staining of primary osteoblasts after stimulation with osteogenic media for 2 weeks as indicated. **i** The corresponding quantitative analysis of ARS staining is based on (**h**). **j** Analysis of osteogenic markers by qRT-PCR after stimulation with osteogenic media for 2 weeks as indicated. *P < 0.05, **P < 0.01, ***P < 0.001.

**Fig. 3.** IFT20 and WWTR1 deficiency in osteoblasts decrease bone formation and increase marrow fat accumulation. **a** Representative H&E-stained images of femur sections from IFT20-KO, WWTR1-KO, dKO mice, and age-matched controls (OSX-663 Cre mice) at 3 months. Scale bars, 100 μm. N=5. **b, c** Quantifying adipocyte number and area in the distal marrow per tissue area based on H&E images. N=5. **d, e** Representative μCT images of decalcified tibiae of 3-month-old IFT20-KO, WWTR1-KO, dKO mice, and age-matched controls after OsO4 staining for 2 hrs as indicated (**d**). Scale bars, 1 mm. N=5. μCT analysis of MAT is at the right panel. N=5. rMAT: regulated MAT; cMAT: constitutive MAT. The qualification of MAT was identified as indicated (**e**). **f** Representative TRAP-stained images of femurs from 3-month-old IFT20-KO, WWTR1-KO, dKO mice and age-matched controls. Scale bar, 200 μm. N=5. **g** The osteoclast numbers were identified as shown. BS: osteoblast surface. N=5. *P < 0.05, **P < 0.01, ***P < 0.001.

**Fig. 4.** IFT20 and WWTR1 synergistically regulate bone homeostasis by activating TGF-β signaling. **a** KEGG analysis of significant change of genes in human osteoporosis. The red box directs to the TGF-β signaling pathway. **b** qRT-PCR analysis of TβRI and TβRII in primary osteoblasts from dKO mice and age-matched controls. Ns: not significant. **c** Western blot analysis of TβRII, Smad2/3, and pSmad2/3 expression in primary osteoblasts isolated from the calvaria of IFT20-KO, WWTR1-KO, dKO mice, and controls. **d** Representative fluorescence images of pSmad2/3 in primary osteoblasts as indicated. Scale bars, 10 μm. **e** Primary osteoblasts from dKO mice were transfected by flag-TβRII or flag empty plasmid for 48 hrs. Then, the cells were induced with the osteogenic medium for 2 weeks. The alizarin red staining was performed as indicated. The intensity of alizarin red staining was measured as shown at right. **P < 0.01. ***P < 0.001.

**Fig. 5.** IFT20 inhibits the ubiquitination and degradation of TβRII via c-Cbl. **a** Primary osteoblasts from the calvaria of wild-type mice were transfected with GFP-IFT20 or GFP empty plasmid for 24 hrs and then treated with 50 μg/mL cycloheximide (CHX) at different times as indicated. The TβRII expression was identified by western blot. **b** Co-IP. After transfection of plasmid GFP-IFT20 or/and flag-TβRII in primary osteoblasts, the interaction of proteins IFT20 and TβRII was identified as indicated. **c** Western blot analysis of TβRII expression demonstrates that TβRII is necessary for TβRII ubiquitination. **d** Western blot analysis of c-Cbl in primary osteoblasts from IFT20-KO mice and controls. **e** After silence of c-Cbl by c-Cbl siRNA in IFT20-overexpressed osteoblasts, the stability of TβRII protein was identified by western blot.

**Fig. 6.** WWTR1 is required for the endogenous expression of TβRII. **a** After silence of TEAD1 using *TEAD1* siRNA in WWTR1-mutant osteoblasts from WWTR1-KO mice, the expression of TβRII was identified by western blot. **b** Schematic binding diagram of WWTR1/TEAD1 complex in *TβRII* promoter. BS and TSS represented the binding site and transcription start site, respectively. **c** ChIP assay. Co-occupation of WWTR1/TEAD1 complex in *TβRII* promoter in primary osteoblasts from WWTR1-KO mice and controls. **d** Co-occupation of WWTR1/TEAD1 complex in *TβRII* promoter after silence of *TEAD1*. **e** IFT20 and WWTR1 synchronously regulate bone homeostasis by modulating the expression and stability of the protein TβRII. **P < 0.01, ***P < 0.001.

**Fig. 7.** IFT20 and WWTR1 deficiency in osteoblasts exacerbate bone-fat imbalance in OVX- and HFD-induced osteoporosis. **a** Representative μCT images of trabecular bones from 3-month-old dKO mice and age-matched controls after 6 weeks of ovariectomy. Scale bars, 1 mm. **b** μCT analysis of OVX samples from (**A**) as indicated. N=5. **c** Representative H&E-stained images of femur sections from OVX samples from (**a**). Scale bars, 200 μm. **d** Quantifying adipocyte number and area in the distal marrow per tissue area based on H&E images. **e** Representative μCT images of decalcified tibiae after OsO4 staining as indicated. Scale bars, 1 mm. **f** μCT analysis of MAT in tibiae from (**e**). **g** Representative μCT images of trabecular bones from 1-month-old dKO mice and age-matched controls after feeding chow and HFD for 3 months. Scale bars, 1 mm. N=5. **h** Bone parameters were identified based on μCT analysis from (Fig. **g**). N=5. **i, j** OsO4 micro-CT staining (**i**) and MAT level (**j**) of decalcified tibiae by μCT analysis as indicated. *P < 0.05, **P < 0.01, ***P < 0.001.
